# An Investigation into the Efficacy of Self-Monitoring in the Context of Mixed Schedules for Reducing Stereotypy in Children with Autism Spectrum Disorders

**DOI:** 10.3390/bs16101905

**Published:** 2026-10-15

**Authors:** Ryan J. Hinderliter, Sarah E. Bloom, Emma R. Devine, Christian N. Spencer

**Affiliations:** Department of Child & Family Studies, College of Behavioral and Community Sciences, University of South Florida, Tampa, FL 33620, USA

**Keywords:** applied behavior analysis, differential reinforcement, behavioral interventions, mixed schedules, self-monitoring

## Abstract

Children with Autism Spectrum Disorder (ASD) may engage in stereotypy (STPY). STPY may be problematic if it interferes with other activities or is self-injurious. We used self-monitoring (SM) alone and in a two-component mixed schedule to reduce stereotypy in three boys with ASD. We used a multiple baseline across participants design with an embedded reversal for one participant. After confirming an automatic function for STPY, baseline was followed by three conditions: Differential Reinforcement (Accuracy), during which the participant received reinforcement for accurately recording STPY; Differential Reinforcement of Other Behavior (DRO) with SM, during which the participant received reinforcement for not engaging in STPY; and a two-component mixed-schedule condition in which the reinforcement contingency (either differential reinforcement for accuracy or the absence of STPY) was unsignaled. Training SM reduced STPY in two participants. However, the third participant’s STPY only reduced with DRO. For him, a two-component mixed schedule in which the reinforcement contingency was unsignaled, reduced STPY, thus reducing the observation requirement for DRO. Thus, using indiscriminable contingencies may contribute to more efficient SM interventions, when reducing STPY is a goal identified by the participant themselves and is considered socially valid.

## 1. Introduction

A frequent target for behavior change in children with Autism Spectrum Disorder (ASD) is stereotypy (STPY), which is a repetitive pattern of behavior and is part of the diagnostic criteria for ASD (American Psychological Association, 2024). STPY is highly prevalent in people with ASD (Chebli et al., 2016). Whether or not it is ethical to employ applied behavior analytic interventions for STPY has been a topic of discussion (e.g., McLaughlin & Fleury, 2018). Practitioners should use behavior reduction procedures exclusively for socially significant behavior (i.e., the behavior is causing impairment of the individual’s well-being). This means refraining from treating STPY that is not interfering with academic progress or other domains important for life.

When deciding whether or not to treat STPY, both the goal and reduction procedures used should also be approved by the participant and others in their life, such as parents and teachers (Heward et al., 2022). This would look like gaining informed assent as well as consent (see Breaux & Smith, 2023, for discussion). However, before a consent form is developed, a behavior analyst must evaluate whether or not they consider the behavior disruptive enough to the individual to merit intervention. Graber and Graber (2023) discussed the necessity for practitioners to distinguish between the appropriate and inappropriate use of STPY reduction procedures. Graber and Graber present three case studies, each of which involved a participant who engaged in STPY. In the first case, the child engages in a topography of STPY that is self-injurious and therefore should be treated. However, the other two case studies present children who engage in STPY that does not cause any form of tissue damage. The cases are different from one another in that one case is engaging in STPY across settings and the STPY is leading to issues at school, whereas in another case the child is only engaging in STPY at home. Graber and Graber recommend that the case that interferes with functioning be treated but that the other case not be treated. A difference between the cases that might be inferred is that in the final case study, the child has learned to engage in discriminated responding and only uses STPY when in a context in which it will not interfere with academic or social goals. Most people engage in some form of stereotypy (Niehaus et al., 2000), but presumably, we have identified topographies that are less disruptive, like leg bouncing, pen tapping, or hair twirling, or have identified ways to engage in topographies that do not interfere with our ability to access other sources of reinforcement. In Graber and Graber’s third case, the differentiated responding across school and home settings was not explicitly taught. But it raises the possibility that children could be taught to identify situations in which STPY is or is not going to be problematic and to restrict STPY to contexts in which it will not disrupt their academic or social efforts. This type of intervention, which teaches someone to engage in discriminated responding, aligns well with previous research on interventions for problem behavior that incorporate the use of multiple schedules (Saini et al., 2016), but with an important addition. Rather than just teach that absence of STPY in one context will be reinforced, this involves the idea that STPY may occur in another context without programmed consequences; this approach adds a feature in which the participant decides when they do or do not feel comfortable engaging in a particular topography of STPY. Thus, the approach engages the possibility for self-determination, which is not necessarily a feature of multiple schedules generally. This could be an example of a client-led approach that recruits an individual to serve as their own behavior-change agent.

Regardless, the question regarding whether or not STPY should be reduced should also include the perspectives of autistic individuals. Chazin et al. (2025) surveyed 660 people, including 226 autistic adults, and found that decreasing STPY and/or restricting STPY to particular contexts were intervention goals ranked very low by respondents, whereas goals associated more directly with harm, such as self-injury and aggression, were ranked much higher. Acknowledging that some STPY overlaps with self-injury, it remains that much STPY is not harmful and should not be targeted. Relatedly, Baiden et al. (2025) asked 235 autistic adults to complete the Autism Intervention Attitudes Scale (AIAS) and found that goals that were associated with improving safety and quality of life were socially valid, whereas goals associated with masking or conforming to neurotypical norms were not. Schuck et al. (2024) report qualitative data that emphasize the importance of autonomy and self-advocacy when considering both behavioral targets and interventions. Therefore, behavior analysts must carefully consider whether or not specific STPY targets are appropriate and should make sure that participants or, in the case of clinical practice, clients are centered in that decision-making process. Beyond assent, including the client in the intervention is one way to ensure that they are in control of what will be targeted and the conditions under which contingencies will be implemented. This may be a way to ensure that outcomes *and* interventions are meaningful and acceptable to the person whose behavior is being changed, as it can serve as a sort of “fail-safe” in that if an intervention (or its target) is unacceptable, an individual can choose not to implement it in their own behavior.

Interventions using a client-led approach might maximize success if they incorporate flexibility and are designed in a way that acknowledges that when a person is their own behavior change agent, their cooperation must be the reinforcer of the intervention designer. In other words, interventions in which one serves as one’s own behavior change agent must be reinforcement-based and uncoercive or they are less likely to be continued. Goldiamond (1974/2002) warns against interventions that require individuals to agree to participate via coercion. He cites Azrin and Powell (1968) as an example of participants deciding not to continue in a smoking cessation study, even though they originally consented, rather than to experience the aversive consequence of shock^1^. Such interventions would need to be reinforcement-based, flexible, and participant-led.

The example described above of such a participant-led approach involves teaching individuals with STPY to differentiate between environments by the presence of discriminative stimuli, to determine when STPY is or is not going to prevent access to other reinforcers. Then, they could engage in STPY in certain contexts but refrain from doing so in others. In addition, they could decide for themselves when it mattered and when it did not. For such an approach to work, a self-monitoring (SM) response (Koegel & Koegel, 1990) would first need to be established.

SM is an approach in which individuals observe and record their own behavior in order to promote self-awareness and behavior change (Dunlap et al., 1991). SM relies on an individual’s participation and so increases the importance of the individual’s assent. This may contribute to the social validity of SM, in treating STPY, with parents or other stakeholders who are interested in increasing a child’s autonomy (Leadbitter et al., 2021).

SM has been used to reduce STPY in individuals with autism spectrum disorder (ASD). Koegel and Koegel (1990) used an SM program to reduce STPY in four participants between the ages of 9 and 14 who all had a diagnosis of autism. In Experiment 1, after a baseline that included whatever STPY reduction methods that were being used by their parents, participants were trained to independently record their STPY behavior. If they recorded the absence of STPY, they received reinforcement, which resulted in reduced STPY. A second experiment promoted generalization with two participants from the first experiment in naturalistic settings (i.e., home and school). The study was an example of an effective self-monitoring treatment package that included minimal caregiver involvement (except for the monitoring of the absence of STPY, necessary to provide reinforcement).

The requirement for the absence of STPY for reinforcement is a sometimes unspoken aspect of SM. This requirement is a differential-reinforcement-of-other (DRO) procedure (Vollmer et al., 2020) that has been used in combination with SM. The combination of SM with DRO may represent a more feasible intervention than DRO alone because the participant does not necessarily need to be watched by another person if their self-recording is accurate. DRO can be challenging because it typically requires constant monitoring to verify the absence of the target behavior (Wilder et al., 2023).

Shabani et al. (2001) replicated Koegel and Koegel (1990), also combining SM and DRO to reduce STPY. They began with discrimination training, in which a 12-year-old boy with ASD was trained to accurately score occurrences and nonoccurrences of his own behavior. He also experienced an SM and DRO condition in which he earned access to preferred items by refraining from engaging in STPY and recording accurately that he was not engaging in STPY. The intervention led to a reduction in STPY, even when the requirement to abstain from STPY was increased to 20 min.

Because both Shabani et al. (2001) and Koegel and Koegel (1990) combined SM with DRO, it is difficult to determine what the relative influence of the SM and DRO components might have been. Fritz et al. (2012) conducted a component analysis of the SM with DRO intervention and had mixed results. The study consisted of two phases: a functional analysis of STPY and a component analysis of the self-monitoring intervention from Koegel and Koegel (1990). The functional analyses demonstrated an automatic function for STPY in all three participants (two adults attending a day treatment program for individuals with intellectual disabilities and one child attending a school for children with exceptional needs). In the second phase, they systematically analyzed the components of the self-monitoring intervention. One participant, Curtis, is of particular interest. During the DR (Accurate) condition in which Curtis earned reinforcers for accurate recording, regardless of whether he engaged in STPY, he engaged in STPY but recorded accurately. In the SM + DRO condition, Curtis recorded accurately and had less STPY. During a DRO-only condition, there was also less STPY. Curtis’s STPY and SM accuracy tracked the contingencies; he only was accurate when to do so was required to meet the contingency, and he only decreased STPY when that was the requirement for reinforcement. The final condition was DRO alone, which suppressed STPY without SM. Thus, in Curtis’s case, it appears that SM was not necessary. However, it may be that had Curtis been unable to determine which reinforcement contingency was in place, he might have done both: recorded accurately and reduced his STPY. A set of conditions in which Curtis would be unable to determine which contingency is in place, as in a mixed schedule of reinforcement (Saini et al., 2016), aligns well with a generalization strategy suggested by Stokes and Baer (1977): indiscriminable contingencies.

Indiscriminable contingencies are contingencies that make it difficult to know if the next response will be reinforced (Cooper et al., 2020). Two ways to establish indiscriminable contingencies that have been researched are delayed reinforcement and intermittent reinforcement (Matson, 2023). In 1984, Baer et al. used delayed reinforcement as an indiscriminable contingency for verbal and nonverbal correspondence training. Four children enrolled in a preschool program were asked what they were going to play before they entered an adjacent playroom. In baseline, the teacher responded “ok” to all of the participants’ responses. In the reinforcement-for-verbalization condition, participants were offered a token for repeating a full sentence stating that they were going to play a game suggested by the teacher. Children did not receive a token if they did not repeat the sentence stating they would play the teacher’s game. In the second experimental condition, children were given reinforcement at the end of the play session for stating they would engage in the play behavior regardless of correspondence during the play session. In the third experimental condition, children were then given reinforcement for correspondence if they engaged in the behavior that they said they would. The results showed that delayed reinforcement with indiscriminable contingencies can be used to program maintenance and generalization after training has ended.

Freeland and Noell (2002) examined delayed reinforcement for programming maintenance in children with ASD. Two third-grade girls experienced the following conditions: baseline, contingent reinforcement, delayed intermittent reinforcement, and a no-reward maintenance condition. The last phase followed the one with delayed intermittent reinforcement and had some things in common with the reward phase, but the children did not receive reinforcers for doing the work. Surprisingly, both participants continued to work well during this phase for a long time, perhaps because the transition from the intermittent-reinforcement to no-reinforcement condition was not salient to the participants. The results echoed Baer et al.’s (1984) findings that indiscriminable contingencies can be used to promote maintenance in delayed reinforcement.

Another way to use indiscriminable contingencies may be through using mixed schedules after multiple schedules. Multiple schedules are a set of reinforcement, punishment, or extinction schedules that alternate based on the passage of time and have a discriminative stimulus associated with each schedule (Saini et al., 2016). A study that sheds some light on how this might be used was conducted by Landa and Hanley (2016) and compared two multiple-schedule options (S+/S− and S+ only) to address high-rate requests for edibles in individuals with autism who persisted in such requests after Picture Exchange Communication System (PECS) training. Stimulus control was achieved effectively, with the schedule involving discriminative stimulus presentation during reinforcement and its removal during extinction (S+ only). Participants attended a special education school, had autism and intellectual disability diagnoses, and engaged in severely problematic behavior. Participants received a communication page with icons representing preferred items, and observers recorded request rates during different components. Results showed that for Jack, requests occurred only in the reinforcement component in the S+ condition, indicating stimulus control. In the mixed condition, indiscriminate responding was observed. For Jack, the S+ arrangement produced the greatest degree of discrimination, with zero rates of requests during extinction components. Participants still made requests in the mixed condition. Therefore, clinicians could use a mixed schedule to maintain behavior without the behavior being reinforced. Although Landa and Hanley’s (2016) purpose was not to show how mixed schedules could maintain desirable behavior, their results suggest that mixed schedules could be used in this way.

The current study extends Fritz et al. (2012) to determine whether using a mixed schedule addresses the issue observed with their participant, Curtis, regarding SM alone not suppressing behavior unless it was combined with DRO for the absence of STPY. In Fritz et al. (2012), Curtis’s STPY only decreased when the signaled contingency for DRO was in place. In the accuracy condition, STPY was maintained. Curtis could record behavior accurately to receive reinforcement during DR (Accurate). For some people for whom signaled contingencies within SM result in accurate recording but continued stereotypic behavior, un-signaled contingencies may allow for continued effectiveness of SM intervention, without DRO. Thus, the DRO could be removed, but the effect it had on response suppression would be kept. If this approach is effective, a possible clinical recommendation would be to use SM with DRO but fade DRO with a mixed schedule. This way, there is no signal for when accuracy is being reinforced compared to when the absence of STPY is being reinforced. From the participant’s perspective, the best way to maximize reinforcement is to not engage in STPY and record accurately.

We replicated the DRO and DR (Accuracy) conditions from Fritz et al. (2012), with a supplementary mixed-schedule condition in which participants received no explicit cues regarding the contingencies and instructions for each component. The primary objective of this study was to examine whether a mixed reinforcement schedule in which contingencies shift between the accuracy of recording and the absence of STPY can reduce STPY for children with ASD. A secondary objective was to examine the relationship between recording accuracy and reductions in STPY.

## 2. Methods

### 2.1. Participants, Target Behaviors, and Setting

The participants were three males between the ages of 6 and 14 years old with a diagnosis of autism who were receiving behavior analytic services and who communicated using age-appropriate vocal language and were able to acquire the skills associated with recording their own behavior. Although not directly evaluated in our project, none of the participants had moderate or severe intellectual disability. All participants had STPY as a behavior targeted for intervention due to its potentially harmful nature or because, when asked, they agreed that they would like to have more awareness of their STPY in order to decide when they want to engage in it and when they do not. Parents expressed concern with their sons’ STPY in social situations with similarly aged peers and in situations when it was difficult to get their attention (such as a busy public space) due to STPY engagement. However, had only parents expressed concern and the participants had responded differently when asked about whether or not they would like to be more aware of when they were engaging in STPY, we would not have included them in the study. It should be acknowledged that although we incorporated an assent process under the supervision of our Institutional Review Board, all participants were minors and there was an inherent power differential between them and the adult researchers.

All participants were able to follow two-step programs and had the motor skills to pick up a pencil and mark a data sheet with a writing utensil. Jack was a 14-year-old male adolescent who engaged in vocal STPY in the form of high-pitched singing and chair rocking. Alex was a 14-year-old male adolescent and engaged in scripting (delayed echolalia of television shows, including “Beavis and Butthead,” “PBS Kids,” and “Frosty the Snowman”). Jim was a 6-year-old male child and engaged in scripting (delayed echolalia of television shows such as “Cars,” “Blippy,” and children’s storybooks like “Don’t Let the Pigeon Drive the Bus,” and “No David”) and finger biting (pressing the teeth down on the fingers or nails). There was no serious tissue damage involved in Jim’s finger biting. Jack and Jim had no prior treatments, and Alex had been shown a visual prompt card for quiet time as a treatment for his STPY. It should be noted that none of the participants in our study had especially harmful topographies of STPY, but because our procedures involved reinforcing accuracy of recording without requiring the absence of STPY, we were concerned about potential adventitious reinforcement of dangerous topographies. We chose these participants because we did not believe they would be injured from their STPY during their participation in our study. The setting for Jack and Jim was the clinic in which they received behavior analytic services, and Alex’s setting was a room in the private school he attended. There was no report that levels of STPY varied across settings for our participants.

### 2.2. Experimental Design and Sequence

The sequence began with preintervention screening, which included the Functional Analysis Screening Tool (FAST, Iwata et al., 2013), to confirm that an automatic function was suspected, and a Multiple Stimulus Without Replacement (MSWO, DeLeon & Iwata, 1996), to identify reinforcers to use during subsequent phases in the study. Next, we began with the baseline, using procedures similar to the autoscreen recommended by Querim et al. (2013) to confirm participants’ stereotypic behavior was automatically maintained. Following the baseline, participants were trained to record and monitor their own behavior accurately. After training was completed, the treatment conditions SM + DRO, DR (Accurate), and the mixed schedule commenced, respectively. The experimental design was a multiple baseline across participants, with an embedded reversal for one participant (Alex).

### 2.3. Materials

Data sheets were used to record the frequency of STPY. A script for describing the SM training procedures was created to give instructions to participants. A timer, data sheets for self-recording, pens, and reinforcers that were indicated by the preference assessment (Oreos ©, Starbursts ^®^, Sour Patch Kids ©, and Cheerios ©) were also included.

#### 2.3.1. Dependent Variable

The dependent variables were the presence of STPY and recording accuracy in SM. The effectiveness of the treatment was indicated by the effect that the independent variable had on the dependent variables (i.e., treatment’s effect on levels of STPY and accurate recording). Decreases in STPY were measured by comparing levels of STPY at baseline with levels of STPY after the intervention.

#### 2.3.2. Response Measurement

Data collectors used paper and pen as described in the materials above to measure the occurrence of STPY with partial interval recording. If the participant engaged in STPY at any point during the interval, the interval was marked as “occurrence” or a check. If no STPY occurred during the interval, then the interval was marked as “nonoccurrence” or an “X”. The intervals in which behavior occurred and did not occur were recorded on a data sheet. The accuracy of self-recording was determined by comparing the researcher’s record to the participant’s record. That is, did the participant record when they engaged in STPY versus when they did not, as compared to the data collector’s record of that same interval?

#### 2.3.3. Interobserver Agreement (IOA)

Video recordings of each session were used by the secondary observer to record data. This secondary observer’s record was then compared to the primary observer’s record to calculate IOA. Data collectors were one advanced undergraduate and one doctoral student from the University of South Florida. Agreement was defined as the data matching between the two observers. Disagreement was defined as the two observers’ data not matching. The percentage of intervals in which STPY was observed was calculated by dividing the intervals where both observers scored the occurrence and nonoccurrence of behavior by the total number of intervals. The smaller number was divided by the larger number of intervals and multiplied by 100%.

For Jack, 56.66% of sessions were scored. IOA was 97.89%, with a range of 90–100%. For Alex, 47.05% of sessions were scored. IOA was 99.37%, with a range of 90–100%. For Jim, 34.76% of total sessions were scored, with a range of 90–100%. Average IOA across all participants was 99.37%.

#### 2.3.4. Treatment Fidelity

A trained observer watched a video recording of baseline, training, and intervention sessions and scored the researchers’ treatment fidelity using a checklist for 25% of sessions. Treatment fidelity was scored based on the degree to which the therapist completed the items listed on the treatment integrity checklist (available from the authors).The treatment integrity checklist included items related to whether the researcher followed the procedures as written (e.g., set the time for the appropriate duration, modeled the behavior when it was time to do so, etc.). The treatment fidelity scores were 99.81% (range 98–100%) for Jack, 99.33% (range 96–100%) for Alex, and 99.04% (range 90.5–100%) for Jim.

### 2.4. Procedures

All sessions occurred in a designated area without competing stimuli that was separate from the participants’ natural environment in the school and clinic setting. There were no designated activities during the sessions beyond the described condition-specific procedures. Therapists responded to bids for attention but did not attempt to engage participants in conversations or place any demands. No leisure materials were available, and the therapist stayed with the participant throughout the sessions. Training and treatment comprised ten 30-s intervals for a total of 5 min per session. Enhanced training, if required, included ten 5-s intervals. Jack participated for 15–45 min per week for 8 weeks. For Alex, participation was 45 min–1 h per day 2–4 days a week for three weeks. For Jim, participation was 15 min–1 h, 1–2 days per week for 7 weeks.

#### 2.4.1. Preference Assessment

A preference assessment of edible items was conducted for each participant. The preference assessment was used to identify reinforcers for training and treatment. The preference assessment was an MSWO with seven items (DeLeon & Iwata, 1996). Preference assessment items were determined by asking caregivers what snacks participants preferred and choosing items that could be delivered and consumed easily during sessions. We chose edible reinforcers as they would not interfere with STPY as some leisure items may have done.

#### 2.4.2. Pre-Intervention Screening

The FAST (Iwata et al., 2013) was used to suggest the function of stereotypic behavior before training and intervention. The FAST is a 16-item questionnaire categorizing antecedent and consequent events related to problem behavior used to suggest possible functions for behavior.

#### 2.4.3. Baseline

Following the FAST, we established the baseline using procedures described by Querim et al. (2013) and used the data to confirm an automatic function.

These sessions were conducted in a contrived environment in a classroom or clinic room free of competing stimuli. The observer did not interact with the participant in any way. There were no programmed consequences following instances of STPY. Sessions consisted of ten 30-s intervals for a total of 5 min per session. The participant and therapist sat at a desk with pens, a timer, and data sheets. The participant did not have any materials. Additional sessions were conducted based on trends in the data as needed to stagger the introduction of SM training for the purposes of the multiple baseline design in order to demonstrate experimental control. Up to 2-min breaks occurred in between sessions, unless the participant requested a break or the bathroom. The resulting data were used as a baseline to compare the other treatment conditions.

#### 2.4.4. Monitoring Training

In monitoring training, participants learned how to report the occurrence and nonoccurrence of a model’s behavior. The model’s behavior was different from the participant’s STPY. The training was implemented until the participant correctly recorded the model’s target behavior for 5 consecutive sessions. The participant and researcher sat at a table using a timer, recording sheets, pens, and edible reinforcers. A researcher acted as a model, and the participant had a data sheet to record behavior. The model engaged in STPY for 30 s, and the participant marked a check to indicate occurrence. When the model did not engage in STPY, the participant marked an X to indicate nonoccurrence. When the timer went off, the researcher prompted the participant to record “check or X”. If the participant marked correctly (regardless of whether or not STPY occurred), praise and a reinforcer were delivered at the end of the interval (e.g., a preferred item identified during the MSWO). If they marked incorrectly, they received feedback and the researcher verbally prompted the participant to mark correctly (e.g., “no, the behavior did happen, mark an X”). The researcher prompted the participant again if the participant did not record a response within 5 s of the timer sounding. The participant recorded their monitoring data on a data sheet. The researcher monitored their performance on a separate data sheet. The participant’s data sheet was compared to the researcher’s data sheet to determine recording accuracy.

#### 2.4.5. Self-Monitoring Training

After learning how to accurately record the model’s STPY, the participants entered self-monitoring training, during which researchers taught the participant to accurately record their own STPY. In these sessions, the participant and therapist sat at a table with a timer, recording sheets, pens, and rewards, just like in Monitoring Training. At the beginning of each session, the therapist set a timer and instructed the participant to record their behavior accurately; when the timer went off, the researcher said “check or X”. If they recorded accurately, they received praise and a preferred edible reinforcer at the end of the interval, regardless of if they engaged in STPY or not. The therapist did not comment on their behavior. If the participant did not record within 5 s of the timer, the therapist reminded them to do it correctly. If they complied, they received praise and a reward. If they did not, the therapist assisted them in recording the correct response, but they did not receive praise or a reward. The participants recorded their self-monitoring behavior on a data sheet while the therapist monitored the participants’ self-recording on a separate data sheet. To assess recording accuracy, the participant’s and therapist’s data sheets were compared, as in the previous condition.

The participants met the criterion to end training if they recorded correctly and independently for five consecutive intervals. Jack and Jim did not meet mastery criteria after 6 sessions (60 intervals) for the monitoring and self-monitoring training. Therefore, they received additional training with more components, which we considered “enhanced training”. The first additional component was a shortening of the modeling trials from 30 s to 5 s. The second component was modeling one nonoccurrence and nine occurrences or one occurrence and nine nonoccurrences. These enhanced procedures resulted in Jack and Jim meeting the mastery criterion.

#### 2.4.6. SM + DRO

The first condition following training was SM + DRO, during which participants recorded occurrence and nonoccurrence of STPY and received reinforcement (praise and an edible item) when STPY did not occur. Participants were given a pen and data sheet. When the timer went off at the end of the 30-s intervals, the researcher said, “check or X”, and the participant would mark either occurrence or nonoccurrence. Participants earned one edible reinforcer at the end of the interval, paired with praise if they did not engage in STPY (e.g., “good job not engaging in STPY”). If participants engaged in STPY, they did not receive reinforcement, even if they recorded accurately. Mastery criteria were reached when participants did not engage in STPY for 90% of intervals for two consecutive sessions, and the number of sessions in the condition was at least three.

#### 2.4.7. Differential Reinforcement (Accurate)

In DR (Accurate), the researchers gave participants reinforcers for accurately documenting whether STPY occurred or did not occur. When the timer went off at the end of the 30 s interval, the researcher said, “check or X”, and the participant marked occurrence or nonoccurrence on a data sheet. Participants received one edible reinforcer at the end of the interval along with praise (e.g., “good job recording accurately”) if they recorded accurately, regardless of whether or not STPY occurred. The session continued until participants accurately recorded their STPY and reached mastery criteria of 90% of intervals for two consecutive sessions and when the number of sessions in the condition was at least three.

#### 2.4.8. Mixed Schedule

During the 2-component mixed schedule, participants experienced half of the intervals in either the SM + DRO or the DR(Accurate) components, as described above. The sessions were divided into ten 30-s intervals. There were five 30-s intervals for each component (SM + DRO and DR [Accurate]). The sequencing of the components was predetermined and semi-random (there were the same number of components for each type) across sessions. The sequencing was determined by using a random number generator. There were no schedule-correlated stimuli. The contingency that was in place (i.e., which component of the mixed schedule was in effect) at any given time was not signaled inadvertently by reinforcing behavior, because edible reinforcement and praise were given at the end of the session, not at the end of each interval. At the end of the 5-min session, participants received praise, and the number of edible reinforcers for which they met the reinforcement criteria (e.g., accurate recording in the DR [Accurate] component and absence of STPY in the DRO component).

### 2.5. Social Validity

The participants’ caregivers were asked to respond to a social validity questionnaire. They were shown videos from the baseline, training, and treatment sessions. The researcher explained the results to them and answered any questions they had, then gave caregivers the questionnaire. The questionnaire was scored on a five-point scale: 1 (strongly disagree), 2 (disagree), 3 (neutral), 4 (agree), and 5 (strongly agree). Caregivers were queried on their opinion of the procedures, if they would recommend the procedures, and if they noticed a continued reduction in their child’s STPY (3 total questions). The participants also received similar questions written in simpler terms meant for children.

## 3. Results

**Figure 1:**
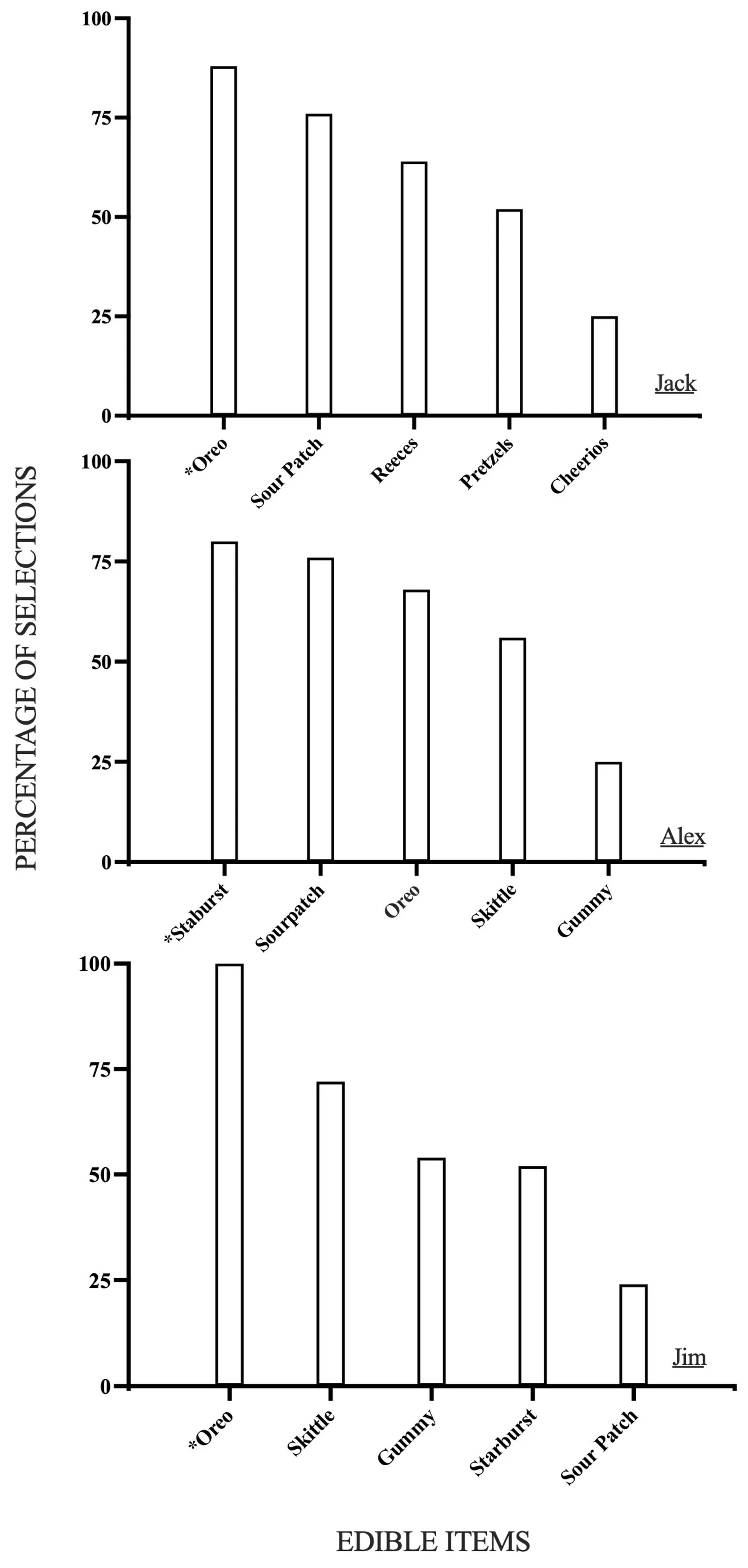
Preference assessment. Graph depicting participant data with the percentage each item was selected when it was available. * Indicates item chosen as participant reinforcer.

The results for the preference assessment (Figure 1) were as follows. Jack showed high preference for Oreos ©, medium preference for Sour Patch Kids ©, Reece’s Pieces ©, and pretzels, and low preference for Cheerios ©. Oreos © were chosen as Jack’s reinforcer. Early on during the study (during SM training), Jack was observed working for Cheerios © outside of the session, and his affect and responding suggested that they were an effective reinforcer. He asked specifically for Cheerios ©, and so his reinforcer was switched from Oreos © to Cheerios © and then was kept consistent throughout the rest of the study. Alex showed high preference for Oreos ©, Sour Patch Kids © and Starbursts ^®^, medium preference for Skittles ^®^, and low preference for gummy candies. Alex selected Starbursts ^®^ and Sour Patch Kids © the most, and so they were used as Alex’s reinforcer. Jim showed high preference for Oreos ©, medium preference for Skittles ^®^, and low preference for gummy candies, Starburst ^®^, and Sour Patch Kids ©. Oreos © were selected as the reinforcer for Jim. For all participants, the delivery of edible reinforcers was paired with praise.

**Figure 2:**
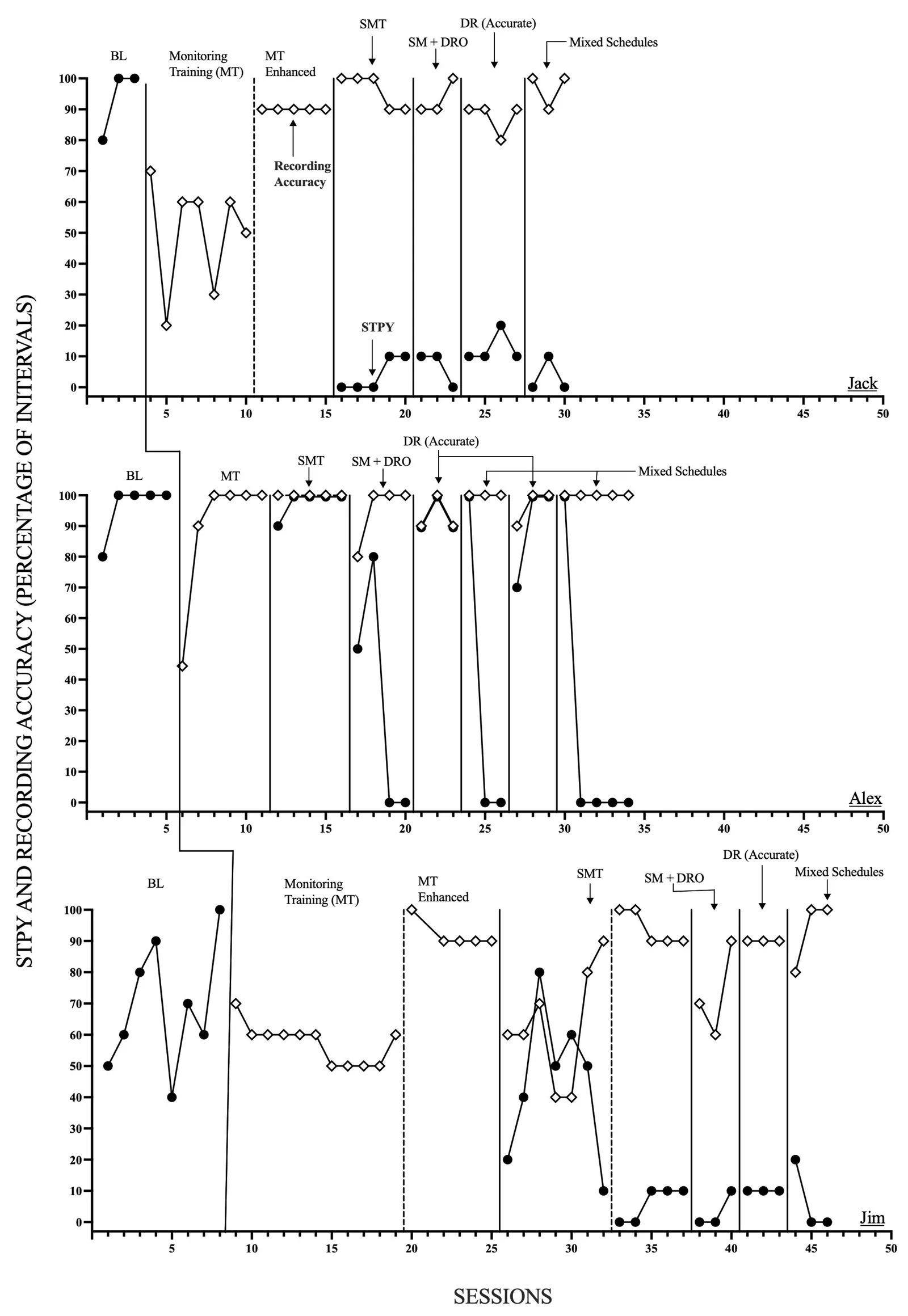
Recording accuracy and STPY. Graphed data from the baseline, monitoring training (MT), self-monitoring training (SMT), SM + DRO, DR (Accurate), and mixed schedules conditions. Open diamonds indicate recording accuracy and closed circles indicate STPY. The vertical dashed line separates the monitoring training from the enhanced monitoring training.

In Figure 2, baseline, both training conditions, and intervention data are graphed. In baseline, all participants engaged in high levels of STPY with an increasing trend, although Jim’s levels were more moderate and variable than Jack’s and Alex’s.

Following the baseline condition, all participants entered monitoring training. During monitoring training, in the first seven sessions, Jack scored 20–70% recording accuracy with variable data. Jack then entered enhanced training (shown after the dotted vertical line in the MT condition), reaching mastery criteria in five sessions with 90% recording accuracy. In monitoring training, Alex increased his recording accuracy from 30 to 100% in six sessions and so did not require enhanced training. During monitoring training, Jim scored between 50 and 70% in recording accuracy for the first 11 sessions, with a slightly decreasing trend. In enhanced training (shown after the dotted vertical line in the MT condition), Jim scored 90–100% recording accuracy.

The self-monitoring training condition followed, and Jack reached the mastery criteria in six sessions, scoring 90–100% accurately. Jack also engaged in STPY between 0 and 10% of intervals. Alex scored 100% recording accuracy and engaged in STPY during 90–100%, with an increasing trend. Jim recorded accurately for 40–80% of sessions, with an increasing trend, and engaged in STPY variably, with a range of 20–80% of sessions during the first seven sessions. In enhanced training, Jim recorded accurately 90–100% of intervals and engaged in STPY 0–10% of intervals.

After training, the intervention condition SM + DRO was introduced. Jack scored between 90 and 100% recording accuracy, with an increasing trend. His STPY levels remained low, ranging from 0 to 10%, with a decreasing trend. Alex had 80–100% recording accuracy, with an increasing trend, and his STPY was variable, ranging from 0 to 80% but ending the condition at 0% of intervals with STPY. Jim increased recording accuracy from 60 to 90% recording accuracy across the condition, and his STPY was between 0 and 10% of intervals.

Following the SM + DRO condition, the DR (Accurate) condition was introduced. Jack’s recording accuracy ranged from 80 to 90%, and his STPY increased slightly from 10 to 20% of intervals with STPY. During the first DR (Accurate) condition, Alex’s recording accuracy was high (90–100%), and his STPY returned to high levels (90–100% of intervals). The DR (Accurate) condition was subsequently reintroduced after the mixed-schedule condition for Alex. During Alex’s second DR (Accurate) condition, his recording accuracy was between 90 and 100%, with an increasing trend, and his STPY ranged from 70 to 100%, with an increasing trend. Jim scored 90% recording accuracy and engaged in STPY during 10% of intervals.

After the DR (Accurate) condition, the mixed-schedule condition was introduced. Jack’s STPY levels remained at a low level, between 0 and 10%. Recording accuracy was 90–100% during the mixed-schedule condition. For Alex, the mixed schedule was introduced, and STPY levels started at 100% in the first session, then dropped rapidly to 0% in the last two sessions, with a decreasing trend. Alex had 100% recording accuracy with this condition. Following the reintroduction of the DR (Accurate) condition, the mixed schedule was reintroduced; STPY levels started at 100% in the first session, then dropped rapidly to 0%. The accuracy of recording was 100% during the second mixed-schedule condition. During the mixed-schedule condition, Jim’s recording accuracy was between 80 and 100%. Jim had STPY levels at 0–10%, with a decreasing trend.

**Figure 3:**
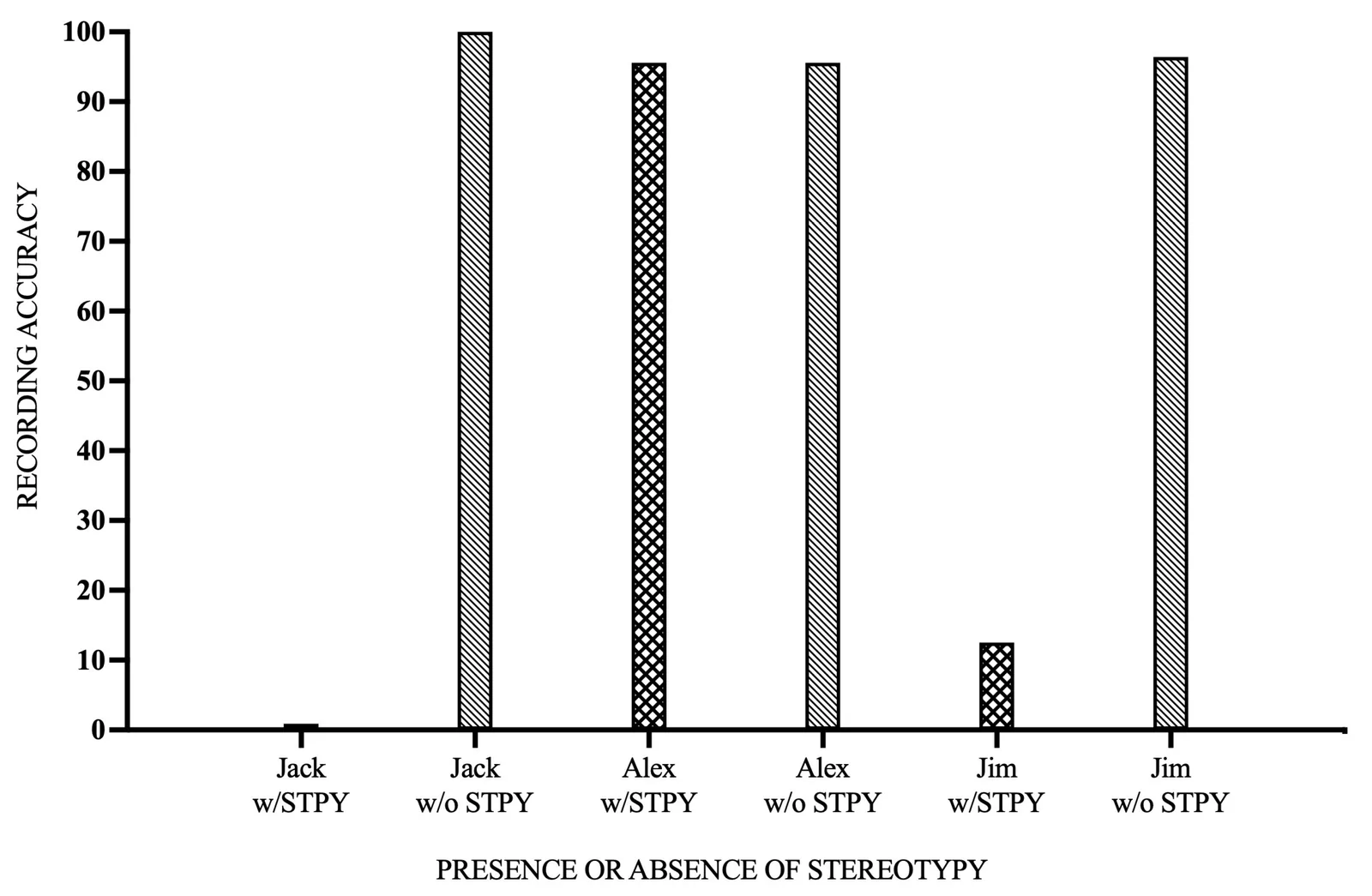
Recording accuracy in the presence or absence of STPY. Graph depicts recording accuracy for intervals with (cross-hatch bars) and without (diagonal-stripe bars) STPY for Jack, Alex, and Jim.

The data for occurrence and nonoccurrence recording accuracy for Jack, Alex, and Jim are graphed in Figure 3. The participants’ correct occurrence recordings and correct nonoccurrence recordings were each divided by the total number of occurrence instances. The results are as follows: Jack recorded 0% occurrences and 100% nonoccurrence intervals correctly, Alex recorded 95.6% of occurrence and 95.6% of nonoccurence intervals accurately, and Jim had 12.5% of occurrence and 96.5% of nonoccurrence intervals recorded accurately.

Social validity results were determined by evaluating questionnaire responses from the participants’ mothers. Jack’s mother “strongly agreed” with the statement, “this treatment for reducing my son’s STPY was something I would like to use at home”. She told the researcher that it would be helpful if the procedures were simplified for parents. Additionally, Alex’s mother strongly agreed with the statement. Jim’s mother gave a three for this question because she also believed the procedures may be too complicated for her to implement at home. Jack, Alex, and Jim’s mother scored “neutral” for noticing a reduction in their sons’ STPY following the experiment. Jack and Jim’s mothers both stated that they had not noticed but also were not looking for it or tracking it. Although they had expressed some concerns about it at the beginning of the study, they did not have any formal data collection systems in place at home and did not feel confident about whether it had decreased in the home setting. Jack, Alex, and Jim’s mothers all strongly agreed they were happy with the way the treatment was conducted with their sons. Jack and Alex’s mothers strongly agreed with the statement that they would recommend this treatment to a friend. Jim’s mother scored “agreed” with this statement again, reiterating that it may be complicated for a friend to implement.

Participants were then asked to fill out the questionnaire. Questions were read aloud, and participants were asked to score the responses from one to five after having received an explanation of the scoring criteria. All participants scored “strongly agree” for the statements “I am ok with the steps that were used during this study”, “I am comfortable reducing my own STPY and monitoring it”, and “this study helped me be aware of my own STPY”. Jack and Alex recorded “strongly agree” for the statement “I will have an easier time not doing my stereotypy if I choose not to in the future as a result of this study”. Jim recorded “disagree” for this statement but did not explain why. Jack, Alex and Jim scored “strongly agree” with the statement “I am happy with the results of the study”.

## 4. Discussion

The present study replicates the DRO and DR (Accuracy) conditions from Fritz et al. (2012), with a supplementary mixed condition in which participants receive no explicit signals to indicate which reinforcement schedule was in place (indiscriminable contingencies). This study builds upon Fritz et al. (2012) to examine whether implementing a mixed schedule can address the issue observed with their participant, Curtis. In their study, Curtis’s STPY decreased only when the signaled contingency for DRO was present. The purpose of this study was to determine if using a mixed schedule with reinforcement contingencies that alternated between accuracy of recording and absence of STPY could effectively reduce STPY for children like Curtis, for whom we only see an absence of STPY when the DRO rule is in place. Only one of our three participants, Alex, exhibited this pattern.

The results showed the mixed schedule was effective for all three participants, but Jack and Jim had other results that were surprising. We saw low levels of STPY during DR (Accurate) for both Jack and Jim, which was unusual, because there was no absence of STPY requirement during that condition. Jack and Jim both engaged in low levels of STPY across all conditions following SM training. Furthermore, in treatment, they were inaccurate when recording STPY occurrence and very accurate when recording no STPY occurrence, as displayed in Figure 3. Jack and Jim’s STPY decreased during self-monitoring training when participants received reinforcement for accurate recording only. The SM training condition was designed to teach self-monitoring, but the results show it also suppressed STPY for these two participants.

It is possible that Jack and Jim engaged in low levels of stereotypy and reported no stereotypy to maximize reinforcement by reducing the effort associated with SM, because if they were not engaging in STPY most of the time, they could just always record no occurrence and maximize reinforcement as long as STPY stayed low. We found this very interesting and hope that Figure 3 clearly shows that only Alex was recording intervals with STPY accurately, whereas Jack and Jim’s global reduction in STPY following training reduced their need to record STPY occurrence. This prompted us to review previous research (Fritz et al., 2012; Koegel & Koegel, 1990; Shabani et al., 2001) to see if their training conditions (upon which we modeled ours) had a similar effect. However, these studies did not display STPY levels in their training data. It is possible that the same effect occurred in their research, but it is unknown because the data are not available. Future studies should include STPY data along with training data to determine the generality of the patterns we observed for Jack and Jim. Regardless, it is promising that for some individuals, simply training them to monitor their STPY results in reductions.

Alex did not reduce STPY in training, nor in any condition in which the absence of STPY was not targeted. Instead, the results showed a pattern like participant Curtis in Fritz et al. (2012). For that participant, recording accuracy was controlled by the rule, but STPY increased when there was no contingency to reinforce the absence of STPY. Meaning, the absence of STPY did not generalize into conditions during which absence was not explicitly reinforced, except during the DR (Accurate) component of the mixed-schedule condition, likely because the contingency was indiscriminable in that condition. During the mixed-schedule condition, Alex’s behavior responded to both the recording accuracy and absence of STPY contingencies, regardless of which was currently in place. In addition, DR (Accurate) was reversed and STPY raised dramatically to high levels after the first implementation of the mixed-schedule condition. This occurred because absence of STPY was no longer reinforced. Once the mixed-schedule condition was reinstated after the DR (Accurate) reversal, STPY decreased substantially.

The results showed that the SM + DRO condition was effective at reducing STPY for three out of three participants, although it was only necessary for one participant. Surprisingly, the DR (Accurate) condition maintained decreased STPY at low levels for two out of three participants. The mixed-schedule condition was effective at reducing STPY for three out of three participants. These findings suggest that using a mixed schedule is as effective as the SM + DRO condition. Additionally, the findings show that using mixed schedules can maintain low levels of stereotypy compared to the DR (Accurate) condition, during which STPY returned to moderate/high levels in Alex’s case. The findings also show that simply reinforcing recording accuracy will cause some participants to reduce STPY, perhaps to maximize reinforcement while minimizing the effort associated with SM.

Our results should be interpreted with the consideration that all three participants were interested in being more aware of their STPY, and different results may be obtained with individuals who were not interested in this. Also, although it was not the only topography of STPY in our study, our participants all engaged in some form of vocal STPY. Vocal STPY might be different than other forms of STPY. In addition, there was some across-participant variation in the timing and duration of conducting sessions. Given that we did not obtain exactly the same results across participants, it could be possible that more uniformity in session scheduling may have resulted in more consistency across results.

Given our results, using mixed schedules during SM might be a useful approach in clinical practice. The implications of these results contribute to the SM and differential reinforcement literature in treating STPY. Additionally, the results contribute to the literature on mixed schedules and their ability to improve treatment outcomes in clinical settings. Mixed schedules, with one component reinforcing accurate recording combined with one reinforcing the absence of STPY, could be applied in various settings to reduce STPY for some individuals and could be used selectively when appropriate and in the context of ethical use of applied behavior analysis.

For example, in a school classroom, when speaking in front of classmates, a student could be taught to gesticulate using their hands when speaking to replace hand flapping. In this example, the behaviors are topographically dissimilar but may serve the same function, but the gesticulating to emphasize spoken points may be more socially appropriate in a peer setting. Without the use of an alternative behavior in this setting, STPY may be socially punished by other children in the form of mocking gestures or comments. In this case, the discriminative stimulus for the alternative response of gesticulating would be speaking to a peer audience. That said, reductions in STPY should be targeted in a manner that honors the dignity and neurodiversity of individuals with ASD (Leaf et al., 2022). Although peers may ostracize children who engage in some forms of STPY, most people engage in at least one form of STPY, albeit one that does not result in social disapproval. For example, Niehaus et al. (2000) found that 98.6% of a sample of 649 college students engage in at least one form of STPY. Stereotypy can be a coping mechanism and may occur more often when an individual is experiencing stress, anxiety, boredom, or excitement or when they are engaging in focused concentration (Shukla & Pandey, 2020). The degree to which people with ASD wish to conform to social norms may vary, and participant assent should always be considered (Breaux & Smith, 2023). However, a means by which people with ASD can learn to self-monitor their own STPY and decide for themselves when or when not to engage in it is a potentially useful aspect of self-determination. Unfortunately, our study is not a demonstration of a fully participant-led approach to addressing STPY, but we hope that researchers and clinicians extend our procedures and develop comprehensive approaches that do incorporate participant decision making regarding when and how to intervene in STPY.

If an autistic person decides that reducing their STPY or changing the contexts in which they engage in it is important to them, clinicians may use these procedures to help them reduce STPY in a clinical setting. Clinicians would first want to discern whether their client is more like Jack and Jim or more like Alex when treating STPY. Clinicians may want to implement the monitoring and self-monitoring training conditions initially and see if STPY levels decrease. If low levels of STPY continue after training, procedures can stop there. This would be helpful to the mothers of our participants, who reported wanting a simpler intervention because it would not involve the use of elements beyond self-monitoring training. If training alone does not maintain low levels of STPY, participants may be more like Alex. For participants like Alex, clinicians may want to train the self-monitoring skills, reinforce the absence of STPY through a DRO, and also reinforce recording accuracy, and then use a mixed schedule of reinforcement so the participant learns to self-monitor and decrease their behavior simultaneously by using indiscriminable contingencies. This can be done by replicating the training and treatment procedures of the current study.

One concern to note is that Jim was offered edible reinforcement for accurate recording and nonoccurrence STPY but denied it nearly every time it was offered. He did receive praise as a reinforcer as well. Because the praise maintained Jim’s low levels of STPY, it appears to have been a reinforcer, and the edible reinforcer may not have been necessary. Although it is typically believed that edible items are more preferred than leisure items (e.g., DeLeon et al., 1997), other research has shown that some children prefer social interaction over edibles (e.g., Lasinski & Klatt, 2024). Future researchers may wish to include non-edible items in preference assessments to identify reinforcers (such as forms of social attention, as in Clay et al., 2013).

Another consideration to note is that in our study, participants received reinforcement at the end of the interval for the training conditions, SM + DRO and the DR (Accurate) conditions. However, in the mixed-schedule conditions, participants received reinforcement at the end of the sessions, rather than at the end of each interval. Future researchers may want to deliver reinforcers at the end of the session rather than individually at the end of the interval for all conditions for a more direct comparison across conditions. However, even with this delay to reinforcement, the mixed-schedule condition was effective for all three participants.

Another limitation that future researchers may wish to address is that we included a therapist prompt at the end of each interval. We did not allow participant behavior to transfer to the timer, and so we do not know whether our participants could engage in self-monitoring without the therapist prompts. Although, anecdotally, we can report that the prompt was not always necessary, we do not have data to report at what point, if any, stimulus control transferred from the therapist prompt to the timer. Future studies may consider procedures that would allow a truly independent self-monitoring mastery criterion.

The social validity questionnaire showed that caregivers approved the procedures. However, two out of three caregivers reported anecdotally that the procedures may be complicated for them to implement in the home. Generalization and maintenance data were not collected for this study, but there was one caregiver questionnaire question about noticing a change in the level of their son’s STPY. All three caregivers recorded “neutral” to that question. The question was more aligned with caregivers’ perception rather than a formal measure of generalization and maintenance. Despite expressing concern for their sons’ STPY levels, two out of three caregivers said their sons’ STPY may have decreased, but they were not watching for a reduction of STPY; thus, the intervention’s effects outside of session are unknown, and future research may wish to assess generalization more directly.

The participants themselves expressed mostly positive responses on the social validity measure; however, these responses should be interpreted with caution because of the inherent power differential between the researchers and participants. The participants were children with autism and may have made positive responses in order to please the researchers. The social validity data we obtained should not be taken as evidence that our procedures do not warrant additional scrutiny. We hope that future researchers will include more meaningful measures of social validity, such as documenting choices to participate on an ongoing basis (anecdotally, none of our participants ever refused a session, but that does not mean that there were no coercive conditions that future researchers should attempt to avoid). We recommend considering Goldiamond’s (1974/2002) arguments regarding coercive contingencies when considering the risks that participants will agree to participate out of acquiescence or because the contingencies strongly favor assent, even when that assent is not meaningful.

Additionally, future research could include training for participants with ASD to determine socially significant settings in which to decrease their own behavior (e.g., school, social events) and measures to determine whether or not participants with ASD found the procedures useful during contexts when they wished to reduce their STPY. This would allow individuals who engage in STPY to be their own behavior-change agent and to determine when it is meaningful for them to reduce their STPY, or to engage in a form of STPY that would not interfere with them accessing other sources of reinforcement, such as peer interactions.

## 5. Conclusions

In conclusion, our study contributes to the literature on self-monitoring and on STPY in multiple ways. It allows a better understanding of what happens to STPY during self-monitoring training by displaying the data and demonstrating that for some individuals, no other reinforcement contingency is necessary to reduce STPY other than the monitoring training. Future studies should continue to include training data in order to better understand the relationship between self-monitoring and behavior. In addition, it presents a solution for those individuals who track contingencies precisely (e.g., Curtis in Fritz et al., 2012, and Alex in the current study) that does not necessarily include constant monitoring as in a traditional DRO. By using indiscriminable contingencies, such as those in the mixed schedule we used, Alex’s STPY decreased even though the DRO contingency was not continuously in place and his monitoring accuracy remained high, even though the accuracy contingency was only present for part of the sessions. This approach may prove useful for behavior analysts who are looking for ways to reduce STPY with self-monitoring interventions without continuous therapist monitoring. Future researchers may wish to extend these results to more naturalistic settings and determine ways to extend the durations of sessions while continuing to use indiscriminable contingencies. As research continues in this area, we hope that others will maintain ethical approaches to STPY, recognizing that STPY is not necessarily always problematic, and will prioritize self-determination for those individuals with ASD who engage in STPY.

## Data Availability

Some deidentified data may be obtained from the authors, as allowed by the IRB and ethical requirements.

## Note

1 It is an interesting aside that the Azrin and Powell (1968) study also included a self-monitoring component.

## Abbreviations

The following abbreviations are used in this manuscript:

ASD	Autism Spectrum Disorder
STPY	Stereotypy
DRO	Differential reinforcement of other behavior
SM	Self-monitoring
